# The Danish Network for Community Pharmacy Practice Research and Development

**DOI:** 10.3390/pharmacy9020114

**Published:** 2021-06-17

**Authors:** Alaa Burghle, Rikke Nørgaard Hansen, Lotte Stig Nørgaard, Ulla Hedegaard, Susanne Bendixen, Lone Søndergaard, Kerly Servilieri, Julianne Hansen, Charlotte Rossing

**Affiliations:** 1 Hospital Pharmacy Funen, Odense University Hospital, 5000 Odense, Denmark; 2 Clinical Pharmacology, Pharmacy and Environmental Medicine, Department of Public Health, University of Southern Denmark, 5000 Odense, Denmark; uhedegaard@health.sdu.dk; 3 Department of Research and Development, Pharmakon, Danish College of Pharmacy Practice, 3400 Hilleroed, Denmark; rnh@pharmakon.dk (R.N.H.); cr@pharmakon.dk (C.R.); 4 Social and Clinical Pharmacy, Department of Pharmacy, Faculty of Health and Medical Sciences, University of Copenhagen, 2100 Copenhagen, Denmark; lotte.norgaard@sund.ku.dk; 5 København Sønderbro Pharmacy, 2300 Copenhagen, Denmark; 199sb@apoteket.dk (S.B.); 199ju@apoteket.dk (J.H.); 6 Aarhus Viby Pharmacy, 8260 Viby J, Denmark; lone.soendergaard@mail.dk; 7 Kløver Pharmacy Brædstrup, 8740 Brædstrup, Denmark; kerly@apoteket.dk

**Keywords:** pharmacy practice, network, pharmacy practice research, research network

## Abstract

The community pharmacy has a number of attributes that makes it an excellent setting for research and development projects, as it is a highly accessible part of the healthcare system and is staffed by highly trained health care professionals. The big turnover in patients in the community pharmacy makes it possible to reach a great number of patients and collect a lot of data in a relatively short time. However, conducting nation-wide research and development projects can be a rather time-consuming process for the individual community pharmacy, and can thus require collaboration with other community pharmacies and researchers. This will help ensure strong results and better implementation. Thus, the Danish Network for Community Pharmacy Practice for Research and Development (NUAP) was established in Denmark by a number of highly committed community pharmacies and researchers. NUAP consists of 102 member pharmacy owners in addition to a number of researchers. The aim of the network is to strengthen pharmacy practice and pharmacy practice research in Denmark by providing a forum where community pharmacy practitioners and researchers meet and work together. The network is led by a steering committee elected by the members in the network.

## 1. Introduction

For the last couple of decades, pharmacy practice has moved from being primarily product-focused to being much more patient-focused [1]. Simultaneously, pharmacy practice research has become an increasingly accepted research field, which has been instrumental in generating evidence for the development of pharmacy health services [2,3,4,5,6,7].

The use of practice-based research networks to collect data and implementation of research results is common, and has been reported in the United States, United Kingdom, Europe, and Canada [8,9,10,11].

The community pharmacy has a number of attributes that makes it a unique and excellent setting for conducting research and development projects. Community pharmacies are highly accessible to patients [12], and are staffed by pharmacists and pharmacy technicians; two groups highly trained in many areas e.g., pharmacology, pharmacotherapy, and communication. Furthermore, the big turnover in customers in the community pharmacy setting [13] makes it possible to collect a large amount of data in a short time [14], and provides a healthcare setting where people in different states of health and illness come to pick up medication and seek medication-related counselling.

However, conducting nationwide research and development projects can be a resource-intensive and time-consuming process for the individual community pharmacy. Collaboration with other pharmacies and researchers might thus be necessary to ensure strong research results and more efficient implementation. Thus, the Danish Network for Community Pharmacy Practice Research and Development (NUAP) was established in Denmark by a number of highly committed community pharmacy owners and researchers in Denmark. NUAP is the abbreviation of Netværk for Udvikling af Apotekspraksis, the Danish name for the network.

In this paper, we will describe the projects included in NUAP and discuss what we have learned throughout the 5 years running the network.

## 2. Community Pharmacies in Denmark

All Danish residents who have been granted a residence permit have access to free healthcare, including visits to a general practitioner, hospital, emergency room, out-of-hour service, and so on. Denmark is divided into 5 regions and 98 municipalities [15]. Managing the hospital system is one of the regions’ main tasks as well as organizing health care services by private practitioners [16], while the municipalities are responsible for the management of health care services at a local level [17].

By October 2020, a total of 506 community pharmacies (including pharmacy branches) exist in Denmark, divided between 201 pharmacy owners [13]. Danish community pharmacies are privately owned and have a monopoly on pharmacy practice, though some over-the-counter (OTC) medications are also sold in retail [18]. The Danish community pharmacy sector is regulated by the state, and is inspected by the Danish Patient Safety Authority and the Danish Medicines Agency along with other health care institutions [15]. On a yearly basis, community pharmacy staff meets the majority of the Danish population—94% of the population visited a community pharmacy in 2017 [19]. Thus, the Danish community pharmacies are responsible for distributing medications and counselling of patients about prescription medication and OTC medication. Additionally, community pharmacies support health promotion and implementation of correct medication use. Danish community pharmacies also deliver other pharmaceutical services for patients [7] e.g., The Inhaler Technique Assessment Service, New Medicines Service, and Re-prescribing Service. Danish community pharmacy staff consist of pharmacists holding a five-year MSc degree in pharmacy, pharmacy technicians holding a three-year academy profession degree, pharmacy technician students, and pharmacy internship students. Owning a community pharmacy is a right reserved for pharmacists in Denmark. An average community pharmacy consists of 12 staff members, while the average number of citizen per pharmacy is 12,000 citizens [13].

A total of 102 pharmacy owners are currently members of NUAP.

## 3. Establishment and Structure of the Network

Denmark has a long tradition for research and development in community pharmacy practice. For decades, a variety of community pharmacy research and development projects have been conducted both locally and nationally [7,20]. However, sharing knowledge and providing support in a structured manner and with full transparency toward stakeholders, researchers, and pharmacy practitioners has proven to be challenging and time-consuming. For this reason, NUAP was established in September 2016 as a network for Danish community pharmacies that are interested in research and development and established Danish researchers in pharmacy practice.

The formal members in the network are the pharmacy owners, but pharmacists or pharmacy technicians from their community pharmacy can represent them in the network. This structure is chosen, as research has found better results in implementation of new initiatives in the community pharmacy when this is supported by a strong leadership of the pharmacy owner [21]. Community pharmacy owners in all five Danish regions can join NUAP. Any community pharmacy owner interested in joining NUAP is required to send an e-mail with a request to join the network to the network secretariat. As a member, you commit yourself to participate in meetings, share knowledge and ideas, and to contribute whichever way you can in projects and dissemination of results.

The network is led by a steering committee that acts as a review board for project ideas submitted to the network by its current and future members. The steering committee consists of up to seven members. Four seats belong to representatives from member community pharmacies, while the remaining three seats belong to representatives from research institutions in the pharmacy practice field. The three research institutions represented are the University of Copenhagen, the University of Southern Denmark, and the Danish College of Pharmacy Practice (Pharmakon). The researchers on the board are appointed by their respective institutions.

The representatives from the community pharmacies serve in the steering committee for a two-year period and are elected by the members at the network’s spring meeting. The four community pharmacy representatives must at least include one pharmacy owner, one pharmacist, and one pharmacy technician.

All projects that aspire to be accepted in the network must be submitted in writing in a defined template project description form for the steering committee to review (Appendix A). The template allows for a maximum of 450 words and covers the following topics: project title, background, aim, methods, timeline, dissemination plan, funding, project manager, and contact information for the project manager and his/her organization (Appendix A).

At present, 10 research and development projects are finalized, and 17 are ongoing.

## 4. The Vision

The overall aim of the network is to strengthen pharmacy practice and pharmacy practice research in Denmark by providing a forum where researchers and representatives of community pharmacies meet to share knowledge and support evidence-based community pharmacy practice. By meeting and working together, the aim of the network is to connect researchers and pharmacy practitioners in Denmark. This is done by creating and cultivating collaborative relationships between community pharmacies and researchers, inspiring new research and development projects, particularly projects rooted in the community pharmacies. Furthermore, the network aims to support transparency in projects and investigations performed in a Danish community pharmacy setting, enhancing project ideas that concern pharmacy practice and emphasize the role of the community pharmacy in relation to the rest of the Danish healthcare system. Additionally, the network aims to carry out projects that are relevant and applicable to future community pharmacy practice. Thus, the collaborative approach between researchers and practitioners enhances both the relevance for practice and the scientific quality of the results gained in the network. The network acts as a facilitator and a platform through which community pharmacies can participate in smaller or larger projects through collaboration with each other.

Finally, the network aims to create and disseminate results from research projects that can emphasize the value of community pharmacy to individual citizens and whole communities.

## 5. How Does the Network Support its Members?

**Figure 1:**
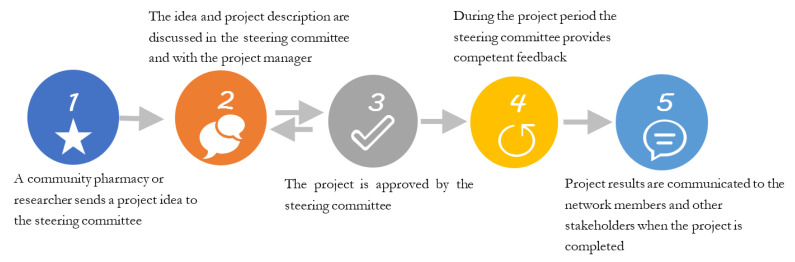
A project’s journey from idea to result dissemination in the Danish Network for Community Pharmacy Practice (NUAP).

The steering committee of the network is ready to provide help and support in projects and project ideas created by the members. Each project is assigned one member of the steering committee to provide support and feedback to the project managers from the community pharmacies (Figure 1).

Further, the steering committee invites all members to a network meeting biannually to discuss new project ideas, developments in ongoing projects, and results from completed projects in the network in addition to other subjects related to pharmacy practice. The biannual network meetings also serve as a forum where members from different community pharmacies from all over Denmark meet, exchange knowledge and experiences, and establish collaborations. The network meetings are conducted either online or as physical meetings. Future meetings will, hopefully, be conducted as a combination of both.

Network members are able to discuss a project idea or a project description with the steering committee at a monthly, open online meeting. Additionally, supporting tools for conducting projects are available on the network’s website e.g., video tutorials about how to carry out a project in a community pharmacy and how to disseminate results.

Participating in the network provides the community pharmacy members with a number of opportunities for inspiration and learning. First, members are, at network meetings and in newsletters, presented with experiences of other members working on different projects. Second, members can share ideas on new pharmaceutical services and other research and development projects that stem from their practice at the community pharmacy. Third, members can participate in a review of practice in the Danish community pharmacies, for which researchers or other project managers ask for input from the practitioners in the network e.g., on how patients with low health literacy or patients who have a poor hearing are supported at the pharmacy. Fourth, the link and collaboration between pharmacy staff and researchers at the universities can contribute to retaining talented and skilled pharmacists and pharmacy technicians in the community pharmacy sector. Last, through participating in the network, community pharmacies have an opportunity to contribute to research and development of pharmacy practice in Denmark and take ownership of the development of their sector along with other community pharmacies and researchers in the field.

Furthermore, members receive help with data processing and dealing with ethical approvals and GDPR regulations from members who are experienced in obtaining such approvals and with working with GDPR (mainly researchers). All projects that involve sensitive personal data are to be approved by either the Regional or National Committee on Health Research Ethics or by the GDPR supervisors in the Danish region in which the project is registered or at one of the universities participating in the project. Both the Committees on Health Research Ethics and GDPR supervisors can waive approval of the project, if they deem such approvals unnecessary. All data collected in research and development projects under NUAP are stored and processed securely, either in secured digital databases or physically in a secure place at the community pharmacies involved in the project in compliance with GDPR regulations.

Some community pharmacies complete and implement projects at their pharmacy all the time, but they seek collaborators for their projects, while other community pharmacies find the network a great opportunity to participate in others’ projects. The network thus creates a win–win situation for both parties.

Community pharmacies and researchers ask for support and competent feedback in different phases of their projects. Thus, the network provides a platform for practitioners and researchers to learn from each other and optimize their research and development projects. The network provides the participating community pharmacies a platform of communicating their results on the network website [22], at network meetings, and in newsletters.

## 6. What Have We Learned So Far?

The members of the network have conducted a large number of studies under a variety of topics and used different methods (Table 1). Some projects have been disseminated through other channels than those displayed in Table 1, e.g., relevant periodicals. Researchers and community pharmacy practitioners alike have initiated projects conducted in the network. Many lessons have been learned through every process involved in the conducted projects, for example:

- The importance of establishing the project group with an allocated project manager to ensure support for the project;
- To ensure that the project yields valid data and robust results, it is important to involve both practitioners and researchers;
- When conducting a project with several community pharmacies participating, it is important to conduct a kick-off meeting with representatives from all participating pharmacies;
- Thorough instructions for the participants and follow up during data collection are important for a successful result;
- Participation in larger projects can enhance the sense of purpose, meaningfulness, and competency for many community pharmacy practitioners and pharmacy practice researchers;
- Participation in research and development projects results in useful knowledge to enhance the daily dialogue and counselling with customers at the pharmacy counter.

The projects in NUAP have a very wide range in both scope and methodology, and have involved both urban and rural community pharmacies from all five Danish regions in all stages of research and development work through projects. Working with the projects in NUAP helps develop and improve pharmacy practice by e.g., involving the community pharmacy in collaborations with other community pharmacies or other stakeholders like municipal homecare to strengthen medication safety for the medicine user. Working within NUAP has made practitioners and researchers work more closely together to research and develop pharmacy practice in Denmark in a way that makes sense in practice.

We hope to read similar reports in the future about relevant initiatives in pharmacy practice from other countries for inspiration and possible future international collaborations.

## 7. What Does the Future Hold for the Network?

The network aims to be the foundation of a strong and national pharmacy practice research environment in Denmark. This can, hopefully, inspire future pharmacy technicians and pharmacists to stay in the community pharmacy sector and to pursue further involvement with the development of the community pharmacy as a platform for delivery of healthcare to the population [7].

The network is now at a stage where dissemination of project results is vital for pharmacy practice and for researchers. The projects are running smoothly and the knowledge generated should be communicated continuously to both researchers and practitioners.

In the future, NUAP seeks to establish international collaborations with other pharmacy practice networks.

## 8. Conclusions

So far, 27 projects have been accepted in the network, of which 10 are finalized.

In conclusion, the network ensures a platform for national knowledge sharing in pharmacy practice, which is advantageous for researchers and practitioners. Researchers have access to a great number of community pharmacies for data collection for small and big research projects, and being part of a vast network gives small community pharmacies the opportunity to contribute to the development of Danish community pharmacy practice on equal terms with larger community pharmacies. This gives the network the potential to become an important Danish development and research resource.

In the future, the steering committee and members of NUAP are interested in working more on integration of the results from projects conducted in the network in all community pharmacies. This can be done by e.g., continuing to work on more practice-based projects that are highly relevant for community pharmacy practice, disseminating results of projects more efficiently both nationally and internationally, and involving more pharmacy technicians as they are the largest staff group in Danish community pharmacies.

In its first five years, the network has experienced a great willingness from Danish community pharmacies to contribute to the development of community pharmacy practice in Denmark. Dissemination of project and research results in the network will increase the visibility of Danish community pharmacy practice and, hopefully, contribute to its development.

## Figures and Tables

**Table 1:** Ongoing and completed projects in the Danish Network for Community Pharmacy Practice (NUAP).

Year	Project Title	Aim	Participants	Data Collection	Dissemination	Project Type	Conclusions
Completed projects							
2017	Reduction in long-term treatment with proton pump inhibitors (PPIs)	To quantify the use of PPI for regular users to determine how many use it regularly and to examine how many have tried to discontinue their PPI-treatment	395 community pharmacy customers	Interviews	Poster at Nordic Social Pharmacy and Health Services Research Conference (NSPC) [23] Video presentation [24]	Development project	A large proportion of PPI users (80%) use their medicine continuously. The leaflet information was a useful tool to provide essential information to new users in addition to counceling at the community pharmacy counter.
2018	Customers’ information seeking behavior prior to community pharmacy visits: a community pharmacy survey	To quantify and describe customers’ information seeking behavior prior to community pharmacy visits, and to describe how pharmacy staff utilize information obtained by customers.	2623 community pharmacy customers	Survey	Burghle et al. [14] Posters at international conferences: Pharmaceutical Care Network Europe (PCNE) and Nordic Social Pharmacy and Health Services Research Conference (NSPC) Video presentation [25]	Descriptive study	A total of 14.4% of customers had sought information prior to visiting the community pharmacy. The majority of customers had used reliable sources, and the information was used during pharmacy counselling.
2018	Interdisciplinary collaboration between community pharmacy and general practitioner	To describe what characterizes the Danish pharmacies’ interdisciplinary collaboration with the general practitioner in the primary sector..	29 community pharmacy staff members	Survey	Master thesis report [26]	Descriptive study	The interdisciplinary collaboration between the pharmacy and the general practitioner in the primary sector was characterized by a high level of ‘trust’, ‘mutual professional respect’, and a clear ‘role distribution’.
2018	Unavailable prescriptions at Danish community pharmacies: A descriptive study	To describe the occurrence and reasons for unavailable prescriptions at Danish community pharmacies as well as the types of drugs involved	2765 prescriptions registered	Survey	Lundby et al. [27] Master thesis by Anne Vejrum Nielsen [28]	Descriptive study	Unavailable prescriptions occur in approximately 1% of all dispensing at Danish community pharmacies. Miscommunication between the patient and general practitioner seems to be the primary source of unavailable prescriptions.
2018	Danish physicians’ and pharmacists’ knowledge, attitude, and behavior regarding antibiotics for children under the age of 15	To describe the knowledge, attitudes, and actions of physicians and community pharmacists regarding antibiotics for children under the age of 15	8 interviews and 762 prescriptions registered	Semi-structured interviews and a cross-sectional survey	Master thesis report [29]	Descriptive and qualitative study	Physicians and pharmacists are aware of the appropriate use of antibiotics and antibiotic resistance. Physicians deviate from guidelines, though, when prescribing antibiotics in regard to choice of antibiotic type and dose.
2018	Roundtable between community pharmacy and homecare to support patient safety	To test roundtable meetings between community pharmacy and homecare staff regarding medication	Four community pharmacies and four municipalities	Surveys and online meeting minutes	Report by Pultz et al. [30] Poster at Pharmaceutical Care network Europe (PCNE)	Development project	The roundtables had a positive influence on quality and safety in medication management and are very relevant for the daily work in homecare. Additionally, homecare staff should be involved in planning the content of the roundtables and prioritizing topics.
2019	Evaluation of the Danish Network for Community Pharmacy Practice Research and Development	To explore the attitudes of community pharmacy staff on the Danish network for Community Pharmacy Practice Research and Development	41 community pharmacy staff	Survey	Report by Julie Valentin [31] Posters at Nordic Social Pharmacy and Health Services Research Conference (NSPC) [32] and at FIP-congress 2018	Descriptive study	Members of the network were generally satisfied with the management of the network. However, they had some suggestions for improvements e.g., and idea bank for their project ideas and a newsletter sent directly to their work e-mail regarding the status of the projects in the network.
2019	Communication between pharmacy staff and older migrants	To investigate the communication between older migrants and pharmacy staff at the community pharmacy counter	152 community pharmacy customers	Survey	Master thesis report [33]	Descriptive study	This pilot study indicates that older migrants with language barriers receive less and more basic medical information at the community pharmacy compared with customers who are Danish native speakers.
2020	COVID-19 at the University of Copenhagen’s internship pharmacies—spring semester 2020	To describe the experiences and reflections of pharmacy internship students from University of Copenhagen about the impact of COVID-19 on Danish community pharmacies	47 pharmacy internship students	Survey	Presentation at FIP virtual conference 2020 [34]	Qualitative study	The students gave, among other things, 12 suggestions for what they think could be maintained after the corona pandemic, e.g., meeting culture, social distancing, training in crisis preparedness.
2020	Experiences with abuse of over-the-counter medication in the community pharmacy	To describe Danish pharmacy pharmacists’ experience in regard to customers and their abuse of over-the-counter medicine	Seven sommunity pharmacists	Semi-structured interviews	Master thesis report [35]	Qualitative study	The pharmacists in this study all had experiences with customers abusing over-the-counter drugs. Pharmacy pharmacist experience abuse of over-the-counter drugs on a weekly basis. Identification of abuse takes place through identification of needs, and attempts are made to deal with it through counseling
**Ongoing projects**							
2016	Improving counselling at the community pharmacy	To explore different ways to improve counselling at the community pharmacy	Pilot study: Two community pharmacies Intervention study: 30 pharmacists and pharmacy technicians from community pharmacies	Literature review Focus group interviews Observational and interview study Test and survey	Kaae et al. [36] Fosgerau, Kaae [37]	Development project	
2017	The pharmacist as a bridge-builder in the transition of care at discharge	To to develop and test a cross-sectoral pharmacist-to-pharmacist service to optimize drug treatment and drug information at sector transitions	6 community pharmacists and clinical pharmacists from hospital	Focus group interviews Individual interviews Documentation of practice via survey	Lech et al. [38] Poster presentation at Nordic Social Pharmacy and Health Services Research Conference (NSPC) [39]	Development project (PhD project)	
2019	Quality of pregnancy prevention in women using teratogenic drugs	To explore patients’ and healthcare professionals’ knowledge about pregnancy prevention in women who use teratogenic drugs	103 patients	Survey	Ongoing	Descriptive study	
2019	Scope and practice of re-prescribing in Danish community pharmacies	To explore practices for the re-prescribing service, with regard to providing input to community pharmacies, the Danish Health Authority, educators, and interest organizations in relation to the status, implementation, and development of the service.	125 re-prescribing situations registered	Survey	Ongoing	Descriptive study	
2020	Data-driven dialogue on medical adherence in Danish community pharmacies with the help of Klikkit’s telehealth solution	To test the KlikKit technology to support patients at three selected pharmacies, and to assess how the overview of patients’ medication use can be included in a consultation about compliance	Ongoing	Focus group interviews	Ongoing	Development project	
2020	Triple whammy effect—safe use of NSAIDs	To explore the number of customers who use the triple whammy combination, including both customers who receive NSAIDs on prescription and customers who buy NSAIDs over the counter. Furthermore, to disseminate awareness of the triple whammy effect among pharmacy staff and customers	Ongoing	Patient survey and documentation of practice Survey for the pharmacy staff	Ongoing	Development project	
2020	Narrative Medicine in pharmacy practice—a feasibility study	To develop a course in narrative medicine for community pharmacists and to investigate the feasibility of the course and the effect of the training on the empathic skills of pharmacists	Ongoing	Educational course in narrative medicine for pharmacists Survey	Ongoing	Development project	
2020	Drug shortages in community pharmacies	To explore the extent of drug shortages in Danish community pharmacies	Ongoing	Survey	Ongoing	Descriptive study	
2020	Instruction of patients in using the positive expriatory pressure (PEP) device at the community pharmacy	To develop the service “Instruction in the use of PEP at community pharmacies in Aarhus”	Ongoing	Observations Interviews	Ongoing	Development project	
2020	Customers’ wishes and attitudes towards the COVID-19 antibody test	To explore the customers’ motivations for taking the test at the community pharmacy, their expectations for the use of the results, and their attitude towards the price of the test	Ongoing	Survey	Ongoing	Development project	
2021	Validation of Living with Medicines Questionnaire	To conduct a translation, cross-cultural adaptation and validation of a Danish version of the Living with Medicines Questionnaire	Ongoing	Translation and cross-cultural adaptation using forward and backward translation	Ongoing	Validation study	
2021	Implementation of screening for streptococcus A at the pharmacy	To explore the attitudes of general practitioners and community pharmacits on implementing a test for streptococcus A at the community pharmacy	Ongoing	Interviews	Ongoing	Qualitative study	
2021	The community pharmacy and cancer-related late effects	To explore how many current and former cancer patients with late effects come to the community pharmacies, their needs and wishes, and whether they are interested in receiving support from a community pharmacist to deal with cancer-related late complications	Ongoing	Survey	Ongoing	Descriptive study	
2021	Patients’ perspectives on the deprescribing process	To explore the patient perspective in the deprescribing process. In addition, the project aims to describe the extent to which and how patients see role of the community pharmacy in the deprescribing process	Ongoing	Interviews	Ongoing	Qualitative study	
2021	Use of pain medication in Denmark: A community pharmacy survey	To explore the use of analgesics among Danish community pharmacy customers	Ongoing	Survey	Ongoing	Descriptive study	
2021	Is the patient information leaflet necessary?	To explore the use of the paper-based patient information leaflets in medication packages by community pharmacy customers vs. the use of digital alternatives	Ongoing	Survey	Ongoing	Descriptive study	
2021	Sustainable conversion of community pharmacies and medicine use in Denmark in a Nordic perspective—a study of the role and perceptions of Danish community pharmacy staff	To examine the Danish community pharmacies in a sustainable context and to describe and understand the community pharmacies’ perception and role in the sustainable development of Danish community pharmacies and the sustainable transition of medicine use in Denmark	Ongoing	Survey Interviews	Ongoing	Descriptive and qualitative study	

## Data Availability

Not applicable.

## Read More

The NUAP website (Danish): https://www.pharmakon.dk/forskning/apoteksnetvaerk/. To contact NUAP, please send an email to the secretariat at paps@pharmakon.dk.

## Appendix A

Project description template.

**Table:**

Project Title:	Triple Whammy—Safe Use of NSAIDs
**Background:**	It is well documented that the combination of diuretics and an ACE inhibitor (or angiotensin II receptor antagonist) with NSAIDs presents a risk of acute renal failure. The combination is known as triple whammy. The combination can result in a 31% increased risk of acute renal failure—the risk is doubled within the first 30 days of treatment with NSAIDs. Together, the three drug groups, used alone or in combination, account for more than half of reported cases of drug-induced acute renal failure. Older adults are especially at risk as kidney function deteriorates with age. As community pharmacy staff meet many patients every day, and possess knowledge about medication, there is a possibility that they can help these customers in their daily counselling, as well as those who may want to buy NSAIDs in retail in the future, without receiving counselling.
**Aim:**	To quantify customers who use the triple whammy drug combination, including both customers who receive NSAIDs on prescription and customers who buy NSAIDs over the counter. To describe the awareness of the triple whammy effect among pharmacy staff and customers. Introducing and implementing an information leaflet on the triple whammy effect, as part of the counselling to these customers.
**Design and methods:**	Information about counselling of all patients who come to the pharmacy to buy any kind of NSAID, both prescription and OTC, will be registered by pharmacy staff. Data collection via digital survey.
**Timeframe:**	January 2020–May 2021
**Dissemination plan:**	Peer-reviewed paper, video presentations, articles in relevant Danish periodicals
**Project manager:**	Anne Mette Jørgensen, Pharmacist at Stege Pharmacy
**Contact information:**	
**Organization:** **(Community pharmacy/researcher etc.)**	Community Pharmacy
**Funding**	U2F fund
**Which member of the steering committee do you wish to work with?**	
